# Riemannian transfer learning based on log-Euclidean metric for EEG classification

**DOI:** 10.3389/fnins.2024.1381572

**Published:** 2024-05-30

**Authors:** Fanbo Zhuo, Xiaocheng Zhang, Fengzhen Tang, Yaobo Yu, Lianqing Liu

**Affiliations:** ^1^The State Key Laboratory of Robotics, Shenyang Institute of Automation, Shenyang, China; ^2^The Institutes for Robotics and Intelligent Manufacturing, Chinese Academy of Sciences, Shenyang, China; ^3^School of Computer Science and Technology, The University of Chinese Academy of Sciences, Beijing, China

**Keywords:** brain-computer interfaces, transfer learning, Riemannian spaces, EEG, motor imagery

## Abstract

**Introduction:**

Brain computer interfaces (BCI), which establish a direct interaction between the brain and the external device bypassing peripheral nerves, is one of the hot research areas. How to effectively convert brain intentions into instructions for controlling external devices in real-time remains a key issue that needs to be addressed in brain computer interfaces. The Riemannian geometry-based methods have achieved competitive results in decoding EEG signals. However, current Riemannian classifiers tend to overlook changes in data distribution, resulting in degenerated classification performance in cross-session and/or cross subject scenarios.

**Methods:**

This paper proposes a brain signal decoding method based on Riemannian transfer learning, fully considering the drift of the data distribution. Two Riemannian transfer learning methods based log-Euclidean metric are developed, such that historical data (source domain) can be used to aid the training of the Riemannian decoder for the current task, or data from other subjects can be used to boost the training of the decoder for the target subject.

**Results:**

The proposed methods were verified on BCI competition III, IIIa, and IV 2a datasets. Compared with the baseline that without transfer learning, the proposed algorithm demonstrates superior classification performance. In contrast to the Riemann transfer learning method based on the affine invariant Riemannian metric, the proposed method obtained comparable classification performance, but is much more computationally efficient.

**Discussion:**

With the help of proposed transfer learning method, the Riemannian classifier obtained competitive performance to existing methods in the literature. More importantly, the transfer learning process is unsupervised and time-efficient, possessing potential for online learning scenarios.

## 1 Introduction

Brain-computer interface (BCI) establishes a direct communication between the brain and external devices, providing a new way for the brain to interact with the external world. It allows humans to interact with their surroundings without the intervention of any peripheral nerve or muscle. BCI extracts brain signals and decodes them into control commands to manipulate external devices (such as wheelchairs) while also provides feedback inputs to the brain, such as visual and electrical stimuli. Consequently, in BCI research, brain signals decoding is indispensable.

There are various ways to collect brain signals, such as electroencephalography (EEG), magnetoencephalography (MEG), and magnetic resonance imaging (MRI). Among these methods, EEG is most easily accepted by both users and practitioners as it has many merits. First, it is non-invasive. EEG signals are obtained by placing multiple electrodes over the scalp of the brain, which reflect its electrophysiological activities. Second, the equipment of EEG is relatively small and thus transportable. Third, the EEG device is relatively cheap and is affordable even for small research groups. Therefore, EEG-based BCI systems hold significant potential for widespread application.

However, EEG signals are non-stationary, non-linear, and characterized by weak amplitudes, low spatiotemporal resolution, low signal-to-noise ratio, and inter-individual diversity, making the decoding of EEG signals very challenging (Lotte et al., 2018). Existing methods for decoding EEG signals can be broadly categorized into three groups, including classical signal processing-based methods, deep learning-based methods, and Riemannian geometry or tensor-based methods (Lotte et al., 2018).

Riemannian geometry-based methods represent EEG signals as covariance matrices, transforming them into the Riemannian space of symmetric positive definite (SPD) matrices. Riemannian classifiers are then established to recognize them. Riemannian geometry-based methods have many advantages over other methods. First, they are robust to noise. Moreover, they are applicable to all commonly used EEG paradigms, including P300, steady state visually evoked potential (SSVEP), and motor imagery (Abiri et al., 2019). Finally, they only require small training samples. Consequently, Riemannian geometry-based methods are a promising group of approaches for brain signal decoding (Congedo et al., 2013).

However, current Riemannian geometry-based methods tend to overlook issues related to changes in data distribution and inter-individual variability. Though these methods exhibit good robustness, taking the factors of data distribution differences into consideration might lead to even better recognition performance.

Data distribution changes often occur in cross-session and cross-subject learning within BCI systems. For instance, the same subject may display distribution disparities resulting from variations in electrode positions or changes in the subject's physiological state between two experimental sessions. Additionally, experiments involving different subjects may be impacted by individual biological distinctions, potentially leading to a decline in performance. To conquer this problem, the standard approach in BCIs involves recalibrating classifiers at the beginning of each experiment through a series of calibration trials. Unfortunately, this approach is a time-consuming process that may make subject fatigue and obtain suboptimal performance since it fails to utilize information from past experiments.

To address above issues, transfer learning is commonly adopted in BCIs (Lotte et al., 2018), which can circumvent the recalibration process. Transfer learning targets to help boost the learning of the task in the target domain using the knowledge in the source domain and the source task (Pan and Yang, 2010). In BCIs, the learning setting of domain adaptation is often encountered, where the feature space between the source domain and the target domain is the same, but the marginal probability distributions of the input data are different.

Multiple domain adaptation methods have been proposed to reduce the distribution divergence (Du et al., 2023; Luo, 2023). Existing domain adaptation methods can be generally divided into three categories, including the sample alignment-based methods, the feature adaptation-based methods, and the deep learning model-based methods (Luo, 2023). The first type of methods try to align the averaged covariance matrix of samples from both the target domain and source domain to the identity matrix and thus brings the marginal probability distributions of input data in two domains closer. The second type of methods leverage mathematical transformations to map the input data from the source domain and target domain into a common feature space, where a classifier trained on transformed source data will generalize well to target data. The deep learning model-based methods use conversational neural networks (CNN) for feature extraction from samples and include feature alignment or adversarial techniques in the training process to encourage the learning of domain-invariant features. However, the feature adaptation-based methods are usually constrained by the feature representations, while deep learning-based method demands high computational resources and imposes stringent requirements on domain discrepancy. Therefore, this study focuses on the sample alignment-based methods.

In the Riemannian framework of sample alignment-based methods, data distribution changes manifest as geometric transformations of covariance matrices, which are referred to as “covariance shift” by He and Wu (2018). To eliminate the shift problem, Zanini et al. (2018) propose to utilize the affine transformation to map the covariance matrices to a reference covariance matrix. Li and Zhang (2019) design a covariance matching approach for semi-supervised domain adaptation. Zheng and Lu (2016) build a personalized EEG-based affective model for transfer learning in an unsupervised manner. Rodrigues et al. (2019) employ procrustes analysis on the SPD Riemannian space, further addressing the covariance shift problem.

In the light of the acknowledged work of sample alignment-based methods, we propose a new transfer learning approach based on procrustes analysis (PA) (Maybank, 2005). PA is a common approach of distribution matching for aligning two data distributions in Euclidean space. It has been extended to Riemannian space for the application of EEG classification, which is termed as Riemannian Procrustes Analysis (RPA) (Rodrigues et al., 2019). However, RPA utilizes affine invariant Riemannian metrics (AIRM), which is computationally intensive. To alleviate the computational burden, this study proposes to use more computationally efficient Log-Euclidean Metric (LEM).

The main contributions in this study are summarized as follows:

- We generalize the Euclidean PA to the Riemannian manifold of SPD matrices equipped with log-Euclidean metric, which is termed as PA-LEM. Due to the nice properties of log-Euclidean metric, the resulted method is equivalent to map the SPD matrices into their logarithm domain and then apply Euclidean PA in the mapped space.
- We augment the RPA with AIRM by LEM to boost the computation of the distribution matching. With LEM, the mean and dispersion of samples are more efficiently obtained. Thus, the proposed RPA with LEM (RPA-LEM) becomes much more computationally efficient.
- We combine the proposed transfer learning approaches with the Riemannian classifier termed as probabilistic learning vector quantization with log-Euclidean metric learning (PLVQ-LEML) (Zhang and Tang, 2022) and validate the performance on two motor imagery EEG datasets. The proposed transfer learning approaches significantly improve the performance of PLVQ-LEML. Compared with RPA, the proposed approach improves the performance of the classifier greater with less computational time.

The remainder of this article is organized as follows. Section 2 briefly introduces the concepts of LEM, PLVQ-LEM, and Procrustes analysis. In Section 3, we derive the proposed methods. Section 4 describes the experiment setup and the results. Section 5 concludes the main contributions of this study.

## 2 Related Work

In this section, we briefly introduce the related concepts of log-Euclidean metric, the Riemannian classifier–probabilistic learning vector quantization with log-Euclidean metric learning (PLVQ-LEML), and the Procrustes analysis that targets for aligning data in Euclidean space.

### 2.1 Log-Euclidean Metric

Riemannian approaches transform the original EEG signal matrices to SPD matrices by calculating the covariance matrices. These SPD matrices live on a curved manifold instead of the flat Euclidean space (Tang et al., 2021a). SPD manifold is a differential manifold equipped with Riemannian metric, defining smoothly varying inner products on tangent spaces. Riemannian metric enables the measurement of angles and lengths of tangent vectors and is crucial for quantifying distances and curves on the manifold.

In the context of EEG classification, two notable metrics are the log-Euclidean metric (LEM) and the affine-invariant Riemannian Metric (AIRM). LEM exhibits many useful properties. For example, it maintains distances invariant under operations such as the matrix inversion, logarithmic multiplication, or orthogonal transformation and scaling (Tang et al., 2023). The basic notations and mathmetical principles of LEM are introduced as follows:

Let 𝕊^+^(*n*) represents the space of all real-valued *n* × *n* symmetric positive definite matrices. 𝕊^+^(*n*) makes a Riemannian manifold if endowed with a Riemannian metric. Log-Euclidean metric (LEM) is a commonly used Riemannian metric on the SPD manifold 𝕊^+^(*n*). It is derived by exploiting the Lie group structure under group operation:


$$\begin{array}{l} {\text{X}}_{1}⊙{\text{X}}_{2}=\exp\left(\log{\text{X}}_{1}+\log{\text{X}}_{2}\right)\text{, for}{\text{X}}_{1},{\text{X}}_{2}\in {𝕊}^{+}\left(n\right) \end{array}$$

where exp represents the matrix exponential function, i.e., $\exp\left(\text{X}\right)=\overset{\infty }{\underset{k=0}{\sum }}\frac{1}{k!}{\text{X}}^{k}=I+\text{X}+\frac{1}{2}{\text{X}}^{2}+\cdots \;$, and log denotes matrix logarithm—the inverse of the matrix exponential function.

The well-studied log-Euclidean metric on Lie group of SPD matrices (Arsigny et al., 2006, 2007) leads to the Euclidean metric in the logarithm domain of SPD matrices. Let 𝕊(*n*) denotes the space of all real-valued *n* × *n* symmetric matrices. For any symmetric matrix **X** ∈ 𝕊(*n*), a one-parameter subgroup in 𝕊^+^(*n*) is defined as


$$\begin{array}{l} \xi \left(t\right)=\exp\left(t\text{X}\right)=\overset{\infty }{\underset{k=0}{\sum }}\frac{1}{k!}{\left(t\text{X}\right)}^{k}=I+t\text{X}+\frac{1}{2}t^{2}{\text{X}}^{2}+\cdots \end{array}$$

with derivative


$$\begin{array}{l} \dot{\xi }\left(t\right)=\text{X}+t{\text{X}}^{2}+\cdots =\text{X}\left(I+t\text{X}+\cdots \;\right)=\text{X}\cdot \exp\left(t\text{X}\right) \end{array}$$

The geodesics are then determined by translated versions of one-parameter subgroup, i.e., γ(*t*) = exp(**V**_1_ + *t***V**_2_) for **V**_1_, **V**_2_ ∈ 𝕊(*n*). Therefore, the geodesic between ${\text{X}}_{1}\in {𝕊}^{+}\left(n\right)$ and ${\text{X}}_{2}\in {𝕊}^{+}\left(n\right)$ is the linear combination in the logarithmic domain:


(1) $$\begin{array}{l} \gamma \left(t\right)=\exp\left(\left(1-t\right)\log{\text{X}}_{1}+t\log{\text{X}}_{2}\right)\\ =\exp\left(\log{\text{X}}_{1}+t\left(\log{\text{X}}_{2}-\log{\text{X}}_{1}\right)\right) \end{array}$$

By definition, the Riemannian exponential map ${\text{Exp}}_{\text{X}}:T_{\text{x}}{𝕊}^{+}\left(n\right)\to {𝕊}^{+}\left(n\right)$ is the mapping that projects a tangent vector **V** to the point on the geodesic at time 1, where the geodesic starts at time 0 from **X** (i.e., γ(0) = **X**) with an initial speed vector **V**, i.e., Exp_**X**_(**V**) = γ(1). By differentiating the geodesic given by Equation 1 at time 0, we obtain the initial speed vector:


$$\begin{array}{l} \dot{\gamma }\left(0\right)=\exp\left(\log{\text{X}}_{1}+t\left(\log{\text{X}}_{2}-\log{\text{X}}_{1}\right)\right)\cdot \left(\log{\text{X}}_{2}-\log{\text{X}}_{1}\right){|}_{t=0}\\ =\exp\left(\log{\text{X}}_{1}\right)\cdot \left(\log{\text{X}}_{2}-\log{\text{X}}_{1}\right)\\ ={\text{X}}_{1}\cdot \left(\log{\text{X}}_{2}-\log{\text{X}}_{1}\right). \end{array}$$

With the initial speed vector **V** = **X**_1_ · (log**X**_2_ − log**X**_1_), the Riemannian exponential map Exp_**X**_1__(**V**) needs to return to the point **X**_2_ on the manifold, i.e., Exp_**X**_1__(**V**) = γ(1) = **X**_2_. We can rewrite **X**_2_ as a function of **V** as follows:


$$\begin{array}{l} {\text{X}}_{2}=\gamma \left(1\right)=\exp\left(\log{\text{X}}_{1}+\left(\log{\text{X}}_{2}-\log{\text{X}}_{1}\right)\right)\\ =\exp\left(\log{\text{X}}_{1}+{\text{X}}_{1}^{-1}{\text{X}}_{1}\left(\log{\text{X}}_{2}-\log{\text{X}}_{1}\right)\right)\\ =\exp\left(\log{\text{X}}_{1}+{\text{X}}_{1}^{-1}\text{V}\right). \end{array}$$

Then, the exponential map induced by the log-Euclidean metric is given as follows:


(2) $$\begin{array}{l} {\text{Exp}}_{{\text{X}}_{1}}\left(\text{V}\right)=\exp\left(\log{\text{X}}_{1}+{\text{X}}_{1}^{-1}\text{V}\right). \end{array}$$

The Riemannian logarithmic map Log_**X**_1__(**X**_2_) is the inverse map of the Riemannian exponential map. It gives the initial speed of the geodesic γ(*t*) starting from points **X**_1_ to **X**_2_, i.e., ${\text{Log}}_{{\text{X}}_{1}}\left({\text{X}}_{2}\right)=\dot{\gamma }\left(0\right)$. Therefore, the logarithmic map induced by the log-Euclidean metric is as follows:


$$\begin{array}{l} {\text{Log}}_{{\text{X}}_{1}}\left({\text{X}}_{2}\right)={\text{X}}_{1}\left(\log{\text{X}}_{2}-\log{\text{X}}_{1}\right). \end{array}$$

The metric at a point on the manifold can be obtained by translating the scalar product on the tangent space at the identity (Arsigny et al., 2007). Let $L_{\text{X}}:{𝕊}^{+}\left(n\right)\to {𝕊}^{+}\left(n\right)$ be the logarithmic multiplication by **X**, that is, for any **A** ∈ 𝕊^+^(*n*), *L*_**X**_(**A**) = exp(log**X**+log**A**) = exp(log**X**)·exp(log**A**) = **X**⊙**A**. The identity matrix **I** ∈ 𝕊^+^(*n*) can be transported to a matrix **X** ∈ 𝕊^+^(*n*) by *L*_**X**_ and any tangent vector Δ at the identity **I** can be transported to a tangent vector **V** at the point **X** ∈ 𝕊^+^(*n*) by the differential of *L*_**X**_, given by *dL*_**X**_(Δ) = **X**Δ. At the identity, the metric is defined as the usual scalar product 〈Δ_1_, Δ_2_〉**_I_** = Tr(Δ_1_Δ_2_). The log-Euclidean metric is required to be invariant by left- multiplication (Arsigny et al., 2007), i.e., 〈**X**Δ_1_, **X**Δ_2_〉_**X**_ = 〈Δ_1_, Δ_2_〉**_I_**, which can only be satisfied with definition:


$$\begin{array}{l} {\langle {\text{V}}_{1},{\text{V}}_{2}\rangle }_{\text{X}}={\langle {\text{X}}^{-1}{\text{V}}_{1},{\text{X}}^{-1}{\text{V}}_{2}\rangle }_{I}=\text{Tr }\left({\text{X}}^{-1}{\text{V}}_{1}{\text{X}}^{-1}{\text{V}}_{2}\right) \end{array}$$

where Tr represents the trace operator. With log-Euclidean metric, the squared geodesic distance between two SPD matrices is given as follows:


$$\begin{array}{l} \delta_{LE}\left({\text{X}}_{1},{\text{X}}_{2}\right)={\langle {\text{Log}}_{{\text{X}}_{1}}\left({\text{X}}_{2}\right),{\text{Log}}_{{\text{X}}_{1}}\left({\text{X}}_{2}\right)\rangle }_{{\text{X}}_{1}}\\ =\text{Tr}\left[{\left(\log{\text{X}}_{2}-\log{\text{X}}_{1}\right)}^{2}\right]. \end{array}$$

which corresponds to a Euclidean distance in the logarithmic domain.

Based on Equations (1, 2), the geodesic emitted at the point **X** in the direction of $\text{V}\in T_{\text{X}}{𝕊}^{+}\left(n\right)$, i.e., γ_*LE*_(0) = **X**, ${\dot{\gamma }}_{LE}\left(0\right)=\text{V}$, can be expressed as follows:


$$\begin{array}{l} \gamma_{LE}\left(t\right)=\exp\left(\log\text{X}+t{\text{X}}^{-1}\text{V}\right). \end{array}$$

### 2.2 PLVQ-LEML

Probabilistic learning vector quantization with log-Euclidean metric learning (PLVQ-LEML) (Zhang and Tang, 2022) is a Riemannian classifier that is designed to classify the data points represented by symmetric positive definite (SPD) matrices. It is an extension of Euclidean robust soft learning vector quantization (Seo and Obermayer, 2003) to deal with such data points taking their non-linear Riemannian geometry into consideration. We will briefly introduce this method here. For more detailed information, please refer to Zhang and Tang (2022).

Consider a data set ${\left\{\left({\text{X}}_{i},y_{i}\right)\right\}}_{i=1}^{m}$, where ${\text{X}}_{i}\in {𝕊}^{+}\left(n\right)$ represents the input data and *y*_*i*_ ∈ 1, ..., *C* denotes the corresponding class label. Here, *C* represents the number of classes. PLVQ-LEML is to learn *M*-labeled prototypes **W**_*j*_, *j* = 1, ..., *M* that locate in the same space as the inputs **X**_*i*_ does, i.e., ${\text{W}}_{j}\in {𝕊}^{+}\left(n\right)$ . The label of the prototype **W**_*j*_ is denoted as *c*_*j*_. Let $W={\left\{\left({\text{W}}_{i},c_{i}\right)\right\}}_{i=1}^{M}$, the marginal probability density function *p*(**X**) that generate the data in the Riemannian space 𝕊^+^(*n*) can be approximated by a Gaussian mixture model:


$$\begin{array}{l} p\left(\text{X}|W\right)=\overset{C}{\underset{y=1}{\sum }}\underset{\left\{j:c_{j}=y\right\}}{\sum }p\left(\text{X}|j\right)P\left(j\right) \end{array}$$

where *P*(*j*) represents the probability that data points are generated by a particular component *j* of the mixture and *p*(**X**∣*j*) denotes the conditional probability that the component *j* generates a particular data point **X**. Here, the conditional probability *p*(**X**∣*j*) is a Gaussian-like probability density function constructed using the Riemannian distance derived from the log-Euclidean metric learning (LEML) framework (Huang et al., 2015), as follows:


$$\begin{array}{l} p\left(\text{X}|j\right)\propto \exp\left(f\left(\text{X},{\text{W}}_{j},Q\right)\right) \end{array}$$

Here,


$$\begin{array}{l} f\left(\text{X},{\text{W}}_{j},Q\right)=-\frac{\delta \left(\text{X},{\text{W}}_{j},Q\right)}{2\sigma^{2}} \end{array}$$

where σ^2^ is a user-defined constant represent the variance, while δ(**X**, **W**_*j*_, *Q*) is the Remainnian distance between **X** and **W**_*j*_ parametrized by a learnable metric tensor *Q* derived from LEML, computed as follows:


$$\begin{array}{l} \delta \left(\text{X},{\text{W}}_{j},Q\right)=\mathrm{Tr}\left[Q\left(\log\text{X}-\log{\text{W}}_{j}\right)\left(\log\text{X}-\log{\text{W}}_{j}\right)\right] \end{array}$$

The metric tensor *Q* is a symmetric semi-definite matrix of size *n* × *n*. Then, the probability density that a data point **X** is generated by the mixture model for the correct class can be given as follows:


$$\begin{array}{l} p\left(\text{X},y|W\right)=\underset{\left\{j:c_{j}=y\right\}}{\sum }p\left(\text{X}|j\right)P\left(j\right) \end{array}$$

With Bayes' theorem, the conditional probability of assigning label *y* to data **X** can be obtained as follows:


$$\begin{array}{l} p\left(y|\text{X};W\right)=\frac{p\left(\text{X},y|W\right)}{p\left(\text{X}|W\right)}=\frac{\sum_{\left\{j:c_{j}=y\right\}}P\left(j\right)\exp\left(f\left(\text{X},{\text{W}}_{j},Q\right)\right)}{\sum_{i=1}^{M}P\left(i\right)\exp\left(f\left(\text{X},{\text{W}}_{i},Q\right)\right)} \end{array}$$

Then, for the dataset ${\left\{\left({\text{X}}_{i},y_{i}\right)\right\}}_{i=1}^{m}$, the likelihood function is as follows:


$$\begin{array}{l} L=\overset{m}{\underset{i=1}{\prod }}p\left(y_{i}|{\text{X}}_{i};W\right)\; \end{array}$$

The prototypes **W**_*j*_, *j* = 1, ..., *M* and the metric tensor *Q* can be learned by minimizing the negative log-likelihood function as follows:


$$\begin{array}{l} \text{E}=-\log\text{L}=\overset{m}{\underset{i=1}{\sum }}\left\{\begin{array}{c} -\log\sum_{\left\{j:c_{j}=y\right\}}P\left(j\right)\exp f\left({\text{X}}_{i},{\text{W}}_{j},Q\right)\\ +\log\sum_{j=1}^{M}P\left(j\right)\exp f\left({\text{X}}_{i},{\text{W}}_{j},Q\right) \end{array}\right\} \end{array}$$

The updating rule of prototypes can be obtained by minimizing above loss function via Riemannian gradient descent algorithm on the Riemannian manifold of SPD matrices equipped with log-Euclidean metric, which is calculated as follows:


$$\begin{array}{l} \log{\text{W}}_{l}\leftarrow \log{\text{W}}_{l}-\\ \frac{\alpha }{\sigma^{2}}\cdot \{\begin{array}{ll} \left(P\left(l|\text{X}\right)-P_{y}\left(l|\text{X}\right)\right)Q\left(\log\text{X}-\log{\text{W}}_{l}\right), & \text{if }c_{l}=y\\ P\left(l|\text{X}\right)Q\left(\log\text{X}-\log{\text{W}}_{l}\right), & \text{if }c_{l}\neq y \end{array} \end{array}$$

where *P*_*y*_(*l*|**X**) and *P*(*l*|**X**) are assignment probabilities:


$$P_{y}\left(l|X\right)=\frac{p\left(l\right)\exp\left(f\left(X,W_{l},Q\right)\right)}{\sum_{\left\{j:c_{j}=y\right\}}p\left(j\right)\exp\left(f\left(\text{X},{\text{W}}_{j},Q\right)\right)},$$

$$P\left(l|X\right)=\frac{p\left(l\right)\exp\left(f\left(X,W_{l},Q\right)\right)}{\sum_{j=1}^{M}p\left(j\right)\exp\left(f\left(X,W_{j},Q\right)\right)}$$

and 0 < α < 1 is the learning rate for prototypes updates.

Similar to the method proposed by Biehl et al. (2015), the updating rule of the metric tensor *Q* obtained by minimizing the cost function via stochastic Riemannian gradient descent algorithm using the quotient geometry with the flat metric in the total space is given as follows:


$$\begin{array}{c} Q\leftarrow \Omega \Omega^{T}\\ \Omega \leftarrow \Omega -\frac{\eta }{\sigma^{2}}\sum_{\left\{j:c_{j}=y\right\}}P_{y}\left(j|\text{X}\right){\left(\log\text{X}-\log{\text{W}}_{j}\right)}^{2}\Omega +\\ \frac{\eta }{\sigma^{2}}\sum_{j=1}^{M}\;P\left(j|X\right){\left(\log\text{X}-\log{\text{W}}_{j}\right)}^{2}\Omega \end{array}$$

where 0 < η < 1 is the learning rate for metric updates.

### 2.3 Procrustes analysis

Procrustes analysis (PA) (Gower and Dijksterhuis, 2004) is a widely used approach to align two data domains in the Euclidean space. Suppose we have two sets of data points that are from the same feature space but drawn from two different distributions, denoted as $X={\left\{x_{i}\in R^{n}\right\}}_{i=1}^{m}$ and $\tilde{X}={\left\{{\tilde{x}}_{i}\in R^{n}\right\}}_{i=1}^{m}$, respectively, there exists a linear relationship between each pair of data points ***x***_*i*_ ∈ ***X*** and ${\tilde{x}}_{i}\in \tilde{X}$ as follows:


$$\begin{array}{l} {\tilde{x}}_{i}-\tilde{m}=dU\left(x_{i}-m\right) \end{array}$$

where $\tilde{m}\in R^{n}$ and ***m*** ∈ *R*^*n*^ represent the centers of the two data sets, respectively, *U* is an orthogonal matrix, representing the rotation between the two data sets, and *d* is a scalar denoting the dispersion difference between the two data sets. The objective of the PA process is to determine the values of $\left\{d,m,\tilde{m},U\right\}$ in order to obtain new data set ${\tilde{X}}^{\left(\text{PA}\right)}$, where ${\tilde{x}}_{i}^{\left(\text{PA}\right)}\in {\tilde{X}}^{\left(\text{PA}\right)}$ perfectly matches ***x***_*i*_. Here, ${\tilde{x}}_{i}^{\left(\text{PA}\right)}$ can be obtained as follows:


(3) $$\begin{array}{l} {\tilde{x}}_{i}^{\left(\text{PA}\right)}=\frac{1}{d}U^{T}\left({\tilde{x}}_{i}-\tilde{m}\right)+m \end{array}$$

Through the process of the transformation 3, ${\tilde{x}}_{i}$ is first to be centralized to zero mean (subtracted $\tilde{m}$), stretched or compressed to unit variance (divided by *d*), then rotated (multiplied by *U*^*T*^), and finally recentralized to mean ***m*** (added by ***m***). The last step that recentralizes ${\tilde{x}}_{i}^{\left(\text{PA}\right)}$ to mean ***m*** is often replaced by centralizing ***x***_*i*_ to zero mean, which is to align two data sets of zero mean.

## 3 Riemannian transfer learning methods based on logarithmic euclidean metric

This section introduces our proposed transfer learning methods that are generalizations of Procrustes analysis to the Riemannian space of SPD matrices using log-Euclidean metric (LEM).

### 3.1 Problem formulation and notation

In this study, the EEG signals are represented in the SPD Riemannian manifold by covariance matrix. The *i*-th trial of EEG signals is presented as follows (Tang et al., 2021b):


$$\begin{array}{l} E_{i}=\left[e\left(t_{i}\right),\ldots ,e\left(t_{i}+l-1\right)\right]\in {\mathbb{R}}^{n\times l} \end{array}$$

where *n* and *l* denote the number of channels and sampled points, respectively. Usually after being bandpass filtered, the signals will become zero mean. Each trial of EEG signals is represented by the sample covariance matrix that can be computed as follows:


(4) $$\begin{array}{l} X_{i}=\frac{1}{l-1}E_{i}E_{i}^{T} \end{array}$$

Then, EEG signals are represented by SPD matrices. The SPD matrice manifests the spatial power distribution of the EEG signals over the brain.

In the case of transfer learning, the source data set $S={\left\{\left({\text{X}}_{i},y_{i}\right)\right\}}_{i=1}^{N_{S}}$ and the target set $T={\left\{\left({\tilde{\text{X}}}_{i},{ỹ}_{i}\right)\right\}}_{i=1}^{N_{T}}$, where ${\text{X}}_{i}\in {𝕊}^{+}\left(n\right)$ and ${\tilde{\text{X}}}_{i}\in {𝕊}^{+}\left(n\right)$ represent the input SPD matrices of the two data sets, respectively, while *y*_*i*_ ∈ {1, ..., *C*} and ỹ_*i*_ ∈ {1, ..., *C*} denote their corresponding class labels, respectively. Here, *C* represents the number of classes.

Suppose the data points are drawn from statistical distributions that can be parameterized solely by their geometric mean and the dispersion around neighboring points. To be more precisely, assume that the underlying statistical distribution generating the data set samples is a mixture of Riemannian Gaussian distributions on the SPD manifold with one mixture for each class (Said et al., 2017).

Under this condition, the statistical information of the source and target data sets can be parameterized by a set consisting of *C* + 2 elements, respectively, as follows:


$$\begin{array}{l} \begin{array}{c} \Theta_{S}=\{\text{M},{\text{M}}_{1},\ldots ,{\text{M}}_{C},d\}\\ \Theta_{T}=\{\tilde{\text{M}},{\tilde{\text{M}}}_{1},\ldots ,{\tilde{\text{M}}}_{C},\tilde{d}\} \end{array} \end{array}$$

Here, **M** denotes the geometric mean of the source data set S, and $\tilde{\text{M}}$ represents the geometric mean of the target data set **T**, which are defined as follows:


$$\begin{array}{l} \begin{array}{c} \text{M}=\text{G}(\{{\text{X}}_{i}|{\text{X}}_{i}\in S\})\\ \tilde{\text{M}}=\text{G}(\{{\tilde{\text{X}}}_{i}|{\tilde{\text{X}}}_{i}\in T\}) \end{array} \end{array}$$

where *G* represents the computation of the geodesic mean. *d* represents the dispersion of the data points **X**_*i*_ around the geometric mean **M** for the source data set S, while $\tilde{d}$ denotes the dispersion of the data points ${\tilde{\text{X}}}_{i}$ around the geometric mean $\tilde{\text{M}}$ for the target data set T. They are computed as follows:


$$\begin{array}{l} \begin{array}{c} d=\underset{{\text{X}}_{i}\in S}{\sum }\delta_{R}^{2}(\text{M},{\text{X}}_{i})\\ \tilde{d}=\underset{{\text{X}}_{i}\in T}{\sum }\delta_{R}^{2}(\tilde{\text{M}},{\text{X}}_{i}) \end{array} \end{array}$$

where $\delta_{R}^{2}\left(\cdot ,\cdot \right)$ represents the squared Riemannian geometric distance function. **M**_*k*_ represents the geometric mean of the data points for a particular class *k* in the source data set S, while ${\tilde{\text{M}}}_{k}$ represents the geometric mean of the data points for a particular class *k* in the target data set T. They are computed as follows:


$$\begin{array}{l} \begin{array}{c} M_{k}=\text{G}\left(\left\{{\text{X}}_{i}|{\text{X}}_{i}\in S\text{and}y_{i}=k\right\}\right)\\ {\tilde{M}}_{k}=\text{G}(\{{\tilde{\text{X}}}_{i}|{\tilde{\text{X}}}_{i}\in T\text{and}{ỹ}_{i}=k\}) \end{array} \end{array}$$

### 3.2 PA-LEM

The log-Euclidean metric constitutes a valid Riemannian metric in the original space of SPD matrices and also provides an equivalence Euclidean metric in the logarithm domain of the SPD matrices. Due to this nice property, a straight forward extension of PA to the Riemannian space of SPD matrices is to perform PA in its logarithm domain.

The mean of the data sets in the logarithm domain can be expressed as follows:


(5) $$\begin{array}{l} \begin{array}{c} \log\text{M}=\frac{1}{N^{S}}\overset{N_{S}}{\underset{i=1}{\sum }}\log\left({\text{X}}_{i}\right)\\ \log\tilde{\text{M}}=\frac{1}{N_{T}}\overset{N^{T}}{\underset{i=1}{\sum }}\log({\tilde{\text{X}}}_{i}) \end{array} \end{array}$$

Similarly, the dispersion *d* or $\tilde{d}$ in the original space corresponds to the variance denoted as *d*′ or $\tilde{d^{\prime }}$ in the logarithm domain, which can be computed as follows:


(6) $$\begin{array}{l} \begin{array}{c} d^{\prime }=\frac{1}{N_{S}}\underset{{\text{X}}_{i}\in S}{\sum }{\left(\log{\text{X}}_{i}-\log\text{M}\right)}^{2}\\ {\tilde{d}}^{\prime }=\frac{1}{N_{T}}\underset{{\tilde{\text{X}}}_{i}\in T}{\sum }(\log{\tilde{\text{X}}}_{i}-\log\tilde{\text{M}}{)}^{2} \end{array} \end{array}$$

Thus, the PA method based on the log-Euclidean metric (PA-LEM) can be formulated as follows:


(7) $$\begin{array}{l} \begin{array}{c} {\left(\log{\text{X}}_{i}\right)}^{\left(\text{PA}-\text{LEM}\right)}=\frac{1}{d}\left(\log{\text{X}}_{i}-\log\text{M}\right)\\ (\log{\tilde{\text{X}}}_{i}{)}^{\left(\text{PA}-\text{LEM}\right)}=\frac{1}{{\tilde{d}}^{\prime }}(\log{\tilde{\text{X}}}_{i}-\log\tilde{\text{M}}) \end{array} \end{array}$$

Rotation is not utilized here as it is found barely improves the performance on the target data set in EEG signal classification in the reference Rodrigues et al. (2019). The algorithm of PA-LEM is summarized in Algorithm 1. It is equivalent to normalize both the source data set and target data set to zero mean and unit variance. After the alignment performed by Algorithm 1, the Riemannian classifier PLVQ-LEML learned in the source data set can be directly applied to the target data set, reducing the learning effort on the target data set.

**Algorithm 1:** PA-LEM.

**Input:** Source data set $S=\left\{\left({\text{X}}_{i},y_{i}\right)\right\},i=1,\ldots ,N_{\text{S}}$ and target data set $T=\{({\tilde{\text{X}}}_{i},{ỹ}_{i})\},i=1,\ldots ,N_{T}$.
**Output:** Source data set $S^{\left(\text{PA-LEM}\right)}$, target dataset $T^{\left(\text{PA-LEM}\right)}$.
1: Project the original data in the logarithmic domain via **X**_*i*_ → log(**X**_*i*_), ${\tilde{\text{X}}}_{i}\to \log\left({\tilde{\text{X}}}_{i}\right)$;
2: Calculate the geodesic centroids log(**M**) and $\log\left(\tilde{\text{M}}\right)$ of both target dataset and source dataset using Equation 5);
3: Calculate dispersions *d* and $\tilde{d}$ using Equation (6);
4: Obtain the transformed source data points ${\left(\log{\text{X}}_{i}\right)}^{\left(\text{PA}-\text{LEM}\right)}$ and target data points $(\log{\tilde{\text{X}}}_{i}{)}^{\left(\text{PA}-\text{LEM}\right)}$ via Equation (7).

### 3.3 PRA-LEM

Riemannian Procrustes analysis (RPA) Rodrigues et al. (2019) is an extension of the Euclidean PA to the Riemannian manifold of SPD matrices equipped with affine-invariance Riemannian metric (AIRM) Zanini et al. (2018). The RPA method involves re-centering, stretching, and rotation based on the intrinsic geometric structure of the SPD manifold. Similar to PA, RPA also needs the computation of Riemannian distance and Riemannian geometric mean induced by AIRM, which involves the inverse of matrices that is very computational demanding. Thus, in this study, to boost the computation, the more computationally efficient LEM is utilized.

Under AIRM, the squared Riemannian distance between two points **X**_*i*_ and **X**_*j*_ in the space 𝕊^+^(*n*) is computed as follows:


$$\begin{array}{l} \delta_{R}^{2}\left(X_{i},X_{j}\right)=\overset{n}{\underset{k=1}{\sum }}\log^{2}\left(\lambda_{k}\right) \end{array}$$

where λ_*k*_ represents the eigenvalues of matrix ${\text{X}}_{i}^{-1}{\text{X}}_{j}$. Note that, here, the compuation of the Riemannian distance induced by AIRM requires matrix inverse which is very time-consuming. The geometric mean of *N* data points {**X**_1_, …, **X**_*N*_} is obtained by minimizing their sum of squared Riemannian distance:


$$\begin{array}{l} \text{G}\left({\left\{{\text{X}}_{i}\right\}}_{i=1}^{N}\right)=\underset{\text{M}\in {𝕊}^{+}\left(n\right)}{\mathrm{argmin}}\overset{N}{\underset{i=1}{\sum }}\delta_{R}^{2}\left(\text{M},{\text{X}}_{i}\right) \end{array}$$

via stochastic Riemannian gradient descent algorithm Moakher (2008). Instead, the Riemannian geometric mean under LEM has closed solution, which is much more computationally efficient.

Here, we present how to adapt the Riemannian Procrustes analysis (RPA) under AIRM to RPA with LEM (RPA-LEM). Since the rotation operation performs poorly in motor imagery EEG signal recognition Rodrigues et al. (2019), we only employ the recentering and stretching operations.

#### 3.3.1 Recenter

The purpose of this step is to recenter the dataset to the identity matrix, which serves as the spatial origin in the SPD manifold. Therefore, the first step of RPA-LEM involves calculating the Riemannian centroid of the original dataset:


(8) $$\begin{array}{l} \begin{array}{c} \text{M}=\exp\left(\frac{1}{N_{S}}\overset{N_{S}}{\underset{i=1}{\sum }}\log\left({\text{X}}_{i}\right)\right)\\ \tilde{\text{M}}=\exp\left(\frac{1}{N_{T}}\overset{N_{T}}{\underset{i=1}{\sum }}\log\left({\tilde{\text{X}}}_{i}\right)\right) \end{array} \end{array}$$

Then, we transform the matrices in S and T so that they are both centered at the identity matrix:


(9) $$\begin{array}{l} \begin{array}{c} {\text{X}}_{i}^{\text{(rct)}}={\text{M}}^{-1/2}{\text{X}}_{i}{\text{M}}^{-1/2}\\ {\tilde{\text{X}}}_{i}^{\left(\text{rcr}\right)}={\tilde{\text{M}}}^{-1/2}{\tilde{\text{X}}}_{i}{\tilde{\text{M}}}^{-1/2} \end{array} \end{array}$$

After re-center, two new datasets become:


$$\begin{array}{l} \begin{array}{c} S^{\left(\text{rct}\right)}=\left\{\left({\text{X}}_{i}^{\left(\text{rct}\right)},y_{i}\right)\text{for}i=1,\ldots ,N_{S}\right\}\\ T^{\text{(rct)}}=\left\{\left({\tilde{\text{X}}}_{i}^{\left(\text{rct}\right)},y_{i}\right)\text{for}i=1,\ldots ,N_{T}\right\} \end{array} \end{array}$$

Note that the law of large numbers from statistics also applies to SPD matrices. If the elements of the dataset are drawn from a statistical distribution with a geometric mean of **M**, as the number of instances *N* increases, the centroid of these *N* matrices will converge to **M**. This implies that in experimental paradigms where experiments are conducted sequentially, it is reasonable to expect that with an increasing number of trials, increasingly accurate estimates of the geometric mean will be obtained.

#### 3.3.2 Stretching

The purpose of this step is to adjust the distributions of the two datasets by stretching them so that their dispersion around the geometric mean becomes equal. According to the Riemannian distance formula based on the affine-invariant metric, this can be achieved as follows:


$$\begin{array}{l} \delta_{R}^{2}\left({\left({\tilde{\text{X}}}_{i}^{\text{(rct)}}\right)}^{s},I_{n}\right)=s^{2}\delta_{R}^{2}\left({\tilde{\text{X}}}_{i}^{\text{(rct)}},I_{n}\right) \end{array}$$

This means that we can adjust the dispersion of $T^{\text{(rct)}}$ by simply moving each matrix along a geodesic path connected to the identity matrix, with the parameter *s* as a scaling factor. We achieve the matching of dispersion between the source and target datasets by stretching the target dataset as follows:


(10) $$\begin{array}{l} {\tilde{\text{X}}}_{i}^{\left(\text{str}\right)}={\left({\tilde{\text{X}}}_{i}^{\left(\text{rct}\right)}\right)}^{s},\;s^{2}=d/\tilde{d} \end{array}$$

where *d* and $\tilde{d}$ are the dispersions of two datasets around their geometric centroids, respectively. Under log-Euclidean metric (LEM), the dispersions can be calculated as follows:


(11) $$\begin{array}{l} \begin{array}{c} d=\underset{X_{i}\in S}{\sum }{\left(\log{\text{X}}_{i}-\log\text{M}\right)}^{2}\\ \tilde{d}=\underset{X_{i}\in T}{\sum }(\log{\tilde{\text{X}}}_{i}-\log\tilde{\text{M}}{)}^{2} \end{array} \end{array}$$

PRA-LEM introduces LEM into the Riemannian Procrustes analysis such that the Riemannian geometric mean and the data dispersions around the Riemannian geometric mean can be more efficiently computed. The computation process of RPA-LEM is summarized in Algorithm 2.

**Algorithm 2:** RPA-LEM.

**Input:** Source data set $S=\left\{\left({\text{X}}_{i},y_{i}\right)\right\},i=1,\ldots ,N_{𝕊}$ and target data set $T=\left\{\left({\tilde{\text{X}}}_{i},{ỹ}_{i}\right)\right\},i=1,\ldots ,N_{S}$
**Output:** Source data set $S^{\left(RPA-LEM\right)}$, target dataset $T^{\left(RPA-LEM\right)}$,
%????? Output
1: Calculate the geodesic centroids **M**_*k*_ and $\tilde{{\text{M}}_{k}}$ of both data sets using Equation (8);
2: Calculate dispersions *d* and $\tilde{d}$ using Equation (11).
3: Re-center the data sets S and T via Equation (9) to obtain the datasets $S^{\left(rct\right)}$ and $T^{\left(rct\right)}$;
4: Stretch the re-centered target data set $T^{\left(rct\right)}$ via Equation 10) to obtain the aligned target data set $T^{\left(RPA-LEM\right)}$, while the corresponding output source data set $S^{\left(RPA-LEM\right)}$ is the re-centered $S^{\left(rct\right)}$.

**Figure 1:**
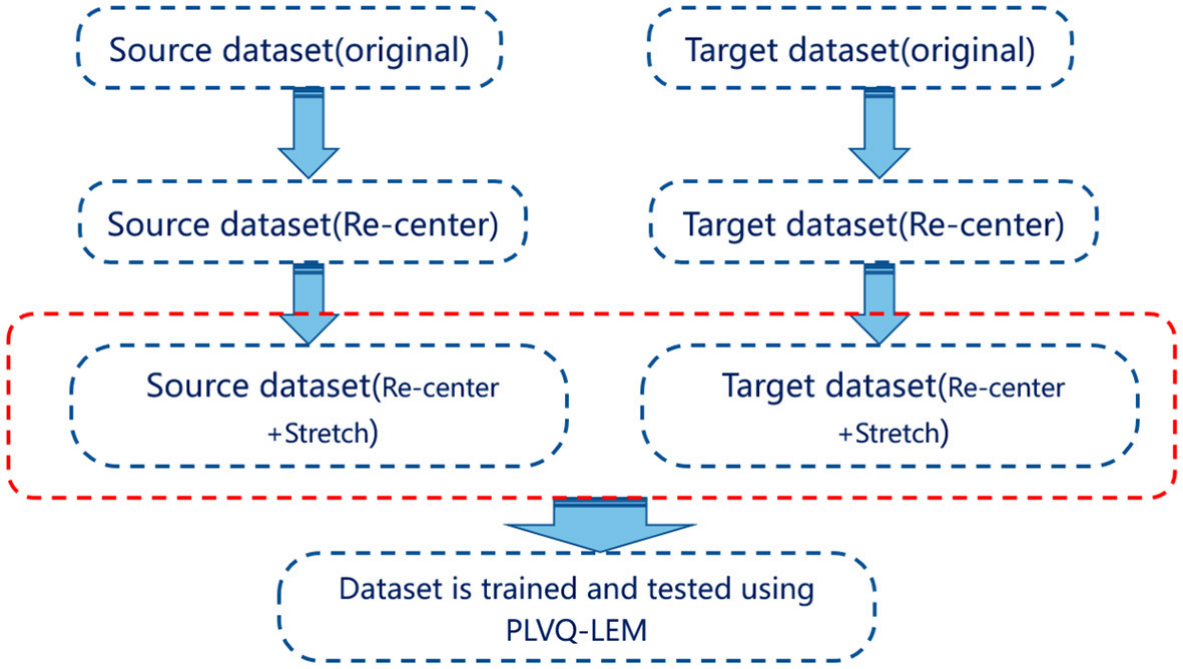
The diagram of Riemannian transfer learning method. Both source dataset and target dataset are processed using re-centering and stretching. Then, the proceeded datasets are utilized for training and testing, respectively.

Figure 1 illustrates the schematic steps of the Riemannian transfer learning method. Note that even though RPA-LEM and PA-LEM use the same way to compute the geometric mean and dispersion, they are inherently not the same. PA-LEM integrates Procrustes analysis (PA) with LEM by transforming the SPD (Symmetric Positive Definite) matrices into the logarithm space. This transformation allows for the application of classical Euclidean computations on the logarithmic domain of the dataset, leveraging the advantages of LEM in handling SPD matrices. However, as the original Euclidean PA did not take account the intrinsic geometry of the SPD manifold, neglecting its geometry-aware nature. Their differences arise from the distinction between LEM and AIRM. LEM emphasizes differences in eigenvalues between matrices, while AIRM emphasizes differences in the overall shape and orientation between matrices. Therefore, RPA-LEM will induce more significant transformations on the original matrix data as compared with PA-LEM. In theory, RPA-LEM should result in better performance. Subsequent experimental results also confirm this.

Additionally, the advantage of computational efficiency brought by log-Euclidean metric mainly comes from the calculation of Riemannian mean and Riemannian distance. In the case of Riemannian mean, the time cost for affine invariant metric is *O*(*K* × *N* × *M*^2^) (assume the average iteration times of Riemannian mean is *K*), while the calculation cost of riemannian mean in log-Euclidean scenario is *O*(*N* × *M*^2^), as the riemmannian mean for LEM is a closed solution. As for the Riemannian distance, the LEM require no inverse operation and matrix multiplication like the AIM does. The time cost for LEM is *O*(*N* × *M*^2^), while the cost for AIM is *O*(*N* × *M*^3^). It is worth noting that these theoretical advantages have also been validated in experiments.

## 4 Experimental result

Two popular motor imagery EEG data sets were used to evaluate our proposed approach, namely, BCI competition III data set IIIa (BCI III IIIa) (Davoudi et al., 2017; Gaur et al., 2018) and BCI competition IV data set 2a (BCI IV 2a) (Tangermann et al., 2012). The two data sets (BCI III IIIa and BCI IV 2a) contain EEG signals extracted using *n* = 60 and *n* = 22 electrodes, respectively, which will be briefly described as follows.

*BCI III IIIa* data set comprises EEG data from three subjects who performed four different motor imagery tasks (left-hand, right-hand, foot, and tongue motor imagery) based on prompts. EEG signals were recorded using a Neuroscan 64-channel EEG signal amplifier with reference electrodes placed on the left and right mastoids, recording from 60 channels, and sampled at 250 Hz. The experiments were repeated across multiple runs (at least 6 runs), with each run containing 10 random presentations of each of the four motor imagery tasks, in total 40 trials per run. Thus, each subject had 240 trials, all conducted on the same date.

*BCI IV 2a* data set comprises EEG data from nine subjects who performed motor imagery tasks involving four types of motor imagery movement tasks including left-hand, right-hand, foot, and tongue motor imagery. Each subject participated in 576 trials, with each trial corresponding to a motor imagery task (144 trials per class). Half of the trials (4 × 72 trials) were conducted in the first session, and the other half (4 × 72 trials) in the second session conducted on different dates. EEG signals from 22 electrodes placed over the sensorimotor cortex of the subjects were recorded at a sampling rate of 250 Hz. During each trial, an arrow cue appeared, pointing left, right, down, or up, corresponding to one of the four classes, to instruct the subject to perform the respective motor imagery task. Motor imagery lasted for 4 s from the appearance of the cue.

For both data sets, recordings of each trial from 0.5 to 2.5 s starting from the presence of the cue were extracted for further analysis. The extracted signals were first bandpass-filtered between 10 and 30 Hz using a 5th-order Butterworth filter. Then, each trial of EEG signals is transformed into an SPD matrice using Equation (4). The inputs of the two datasets become elements of which live in 𝕊^+^(22) and 𝕊^+^(60), respectively.

In this study, *Kappa* coefficient, one of the most commonly used metric to measure the performance of motor imagery EEG classification, was used. It effectively penalizes model bias and serves well for both consistency checks, evaluating classification effectiveness. For data of balanced classes involved in this study, *Kappa* is calculated as *Kappa* = (*D* − *C*)/(1 − 1/*C*), where *D* denotes classification accuracy, and *C* signifies the number of classes.

### 4.1 The choice of classifier and transfer strategies

To evaluate our proposed Riemannian transfer learning methods, a Riemannian classifier is needed. In this study, we chose our previously developed PLVQ-LEML method introduced in Section 2.2 as the Riemannian classifier. This classifier is selected mainly due to the following three considerations:

- The PLVQ-LEML classifier is developed based on Riemannian geometry, targeting to deal with data living on the manifold of SPD matrices. The proposed Riemannian transfer learning methods are also developed based on Riemannian geometry, in particular, geometric transformations that align distributions for data represented by points living on the manifold of SPD matrices. Thus, the combination of PLVQ-LEML with our proposed Riemannian transfer learning methods ensures compatibility and a natural extension of interpretability.
- The PLVQ-LEML classifier is developed using log-Euclidean metric, whose learning rule of prototypes is equivalent to perform the Euclidean updating rule in the logarithm domain. This enables an effective integration with the PA-LEM method.
- Due to the efficiency of log-Euclidean metric, the computation of PLVQ-LEML is very efficient compared with other Riemannian classifiers. The high computational efficiency provides the method higher potential to be used in BCI systems.

We performed a preliminary experiment to compare the classification performance of the PLVQ-LEML with minimum distance to Riemannian mean (MDRM), another computationally efficient Riemannian classifier.

All hyper-parameters were set following the same schedule as mentioned in the study by Zhang and Tang (2022). During training process, the learning rates are set to be decreased over iterations. The learning rate of the prototypes follows the annealing schedule $\alpha \left(t\right)=\frac{n\xi }{100}0.01^{t/T}$, where *n* is the rank of the input matrix, ξ represents the number of prototypes per class, and *T* represents the training epochs. The learning rate for the distance matrix tensor *Q* follows the annealing schedule $\eta \left(t\right)=\frac{n\xi }{10,000}0.01^{t-t_{0}/T-t_{0}}$, where *t*_0_ is the start iteration index for updating the distance matrix *Q*. In order to stabilize the training process, η(*t*) is set to be smaller than α(*t*) and the distance matrix tensor was started to learn when the learning of the prototypes was table. *t*_0_ = 1 was used in this study.

The hyper-parameters *N* and σ^2^ were selected from {1, 2, 3, 4, 5, 6, 7, 8} and {0.5, 1, 1.5, 2, 2.5, 3, 3.5, 4}, respectively, using five-fold cross validation on the training fold. The selected optimal hyper-parameters are shown in Tables 1, 2. The detailed information can be found in our previous published study of PLVQ-LEML Zhang and Tang (2022). Table 3 presents the averaged kappa values over all subjects within the dataset BCI IV 2a and BCI III IIIa. From Table 3, we can observe that the performance of PLVQ-LEML is much better than MDRM. Taking both classification performance and computationally efficiency into consideration, we choose PLVQ-LEML as the classifier in this study.

**Table 1:** Selected hyperparameters of PLVQ-LEML on BCI III IIIa data set.

**Parameters**	**Subjects**		
	k3b	k6b	l1b
*N*	1	2	1
σ^2^	5	4.5	4.5

*N* represents the numbers of prototypes for each class in PLVQ-LEML.

**Table 2:** Selected hyperparameters of PLVQ-LEML on BCI IV IIa data set.

**Para- meters**	**Subjects**								
	**S1**	**S2**	**S3**	**S4**	**S5**	**S6**	**S7**	**S8**	**S9**
*N*	1	2	1	1	1	2	2	1	1
σ^2^	1.5	2	2	2.5	2	1.5	6	1.5	3

*N* represents the numbers of prototypes for each class in PLVQ-LEML.

**Table 3:** Averaged kappa of PLVQ-LEML compared with that of MDRM.

**Method**	**BCI IV 2a**	**BCI III IIIa**
PLVQ-LEML	**0.5885**	**0.7025**
MDRM	0.5200	0.6222

The best performance is marked in boldface.

We also performed a preliminary experiment to compare the transfer strategies. As we mentioned in Section 3, our transfer strategies involve two operations, namely, re-center (rct) and stretching (str). We compared the performance of the transfer learning approaches under different conditions, including using only re-center and combination of re-center and stretching in cross-session transfer setting. Tables 4, 5 demonstrate the performance comparison between the proposed method and RPA under different conditions on both BCI IV 2a and BCI III IIIa data sets. From Tables 4, 5, we can observe that the performance of re-center only performs slightly better than combining re-center with stretching operation. This also confirm the findings in the study by Rodrigues et al. (2019). Thus, in our following experiments, our proposed PA-LEM and RPA-LEM, together with the original RAP, only use re-center operation.

**Table 4:** Performance comparison between the proposed method and RPA under different conditions on the data set BCI IV 2a.

	**RPA**		**PA-LEM**		**RPA-LEM**	
**Subject**	**Rct**	**Rct + str**	**Rct**	**Rct + str**	**Rct**	**Rct + str**
S1	**0.8244** (0.004)	0.8222 (0.005)	**0.8009** (0.012)	0.6759 (0.009)	0.8250 (0.008)	**0.8269** (0.006)
S2	0.3306 (0.007)	**0.3312** (0.014)	**0.3240** (0.032)	0.1620 (0.004)	**0.3293** (0.006)	0.3278 (0.005)
S3	**0.8537** (0.004)	0.8528 (0.003)	**0.8565** (0.009)	0.736 (0.008)1	0.8503 (0.003)	**0.8515** (0.004)
S4	0.5373 (0.006)	**0.5367** (0.007)	**0.5046** (0.005)	0.3519 (0.004)	**0.5417** (0.009)	0.5395 (0.006)
S5	0.3565 (0.011)	**0.3586** (0.008)	**0.3564** (0.012)	0.2407 (0.007)	**0.3596** (0.008)	0.3583 (0.009)
S6	**0.3802** (0.008)	0.3765 (0.008)	**0.3055** (0.010)	0.2731 (0.012)	**0.3799** (0.009)	0.3790 (0.004)
S7	**0.8105** (0.006)	0.8099 (0.007)	**0.8009** (0.011)	0.6898 (0.09)	**0.8105** (0.008)	0.8096 (0.004)
S8	0.7728 (0.005)	**0.7756** (0.005)	**0.7453** (0.003)	0.6481 (0.005)	**0.7799** (0.008)	0.7769 (0.007)
S9	0.7596 (0.007)	**0.7611** (0.006)	**0.7222** (0.007)	0.7037 (0.009)	**0.7707** (0.006)	0.7691 (0.008)
mean	**0.6251**	0.6250	**0.6018**	0.4979	**0.6274**	0.6265

The better performed condition for each method is marked in boldface.

**Table 5:** Performance comparison between the proposed method and RPA under different conditions on the data set BCI III IIIa.

	**RPA**		**PA-LEM**		**RPA-LEM**	
**Subject**	**Rct**	**Rct+str**	**Rct**	**Rct+str**	**Rct**	**Rct+str**
k3b	**0.9200** (0.016)	0.9188 (0.012)	**0.9192** (0.022)	0.9179 (0.027)	**0.9259** (0.019)	0.9241 (0.021)
k6b	0.4736 (0.015)	**0.4689** (0.013)	**0.4593** (0.014)	0.4559 (0.014)	**0.4778** (0.034)	0.4712 (0.025)
l1b	0.7411 (0.027)	**0.7439** (0.032)	0.7333 (0.023)	**0.7348** (0.029)	**0.7444** (0.019)	0.7416 (0.031)
mean	**0.7116**	0.7105	**0.7039**	0.7029	**0.7160**	0.7123

The better performed condition for each method is marked in boldface.

### 4.2 Visualization

To better understand the issue in classification using cross-session or cross-subject EEG data, we visualized the data using the t-distributed stochastic neighbor embedding (t-SNE) technique. t-SNE maps data points in a high-dimensional space to a lower dimensional space while preserving the pairwise distances of the data points as much as possible. Thus, the utilization of t-SNE techniques can well preserve the Riemannian distances between data points that live on the Riemannian manifold of SPD matrices. Here, we project the data points on the Riemannian manifold of SPD matrices into a two-dimensional space for visual inspection.

**Figure 2:**
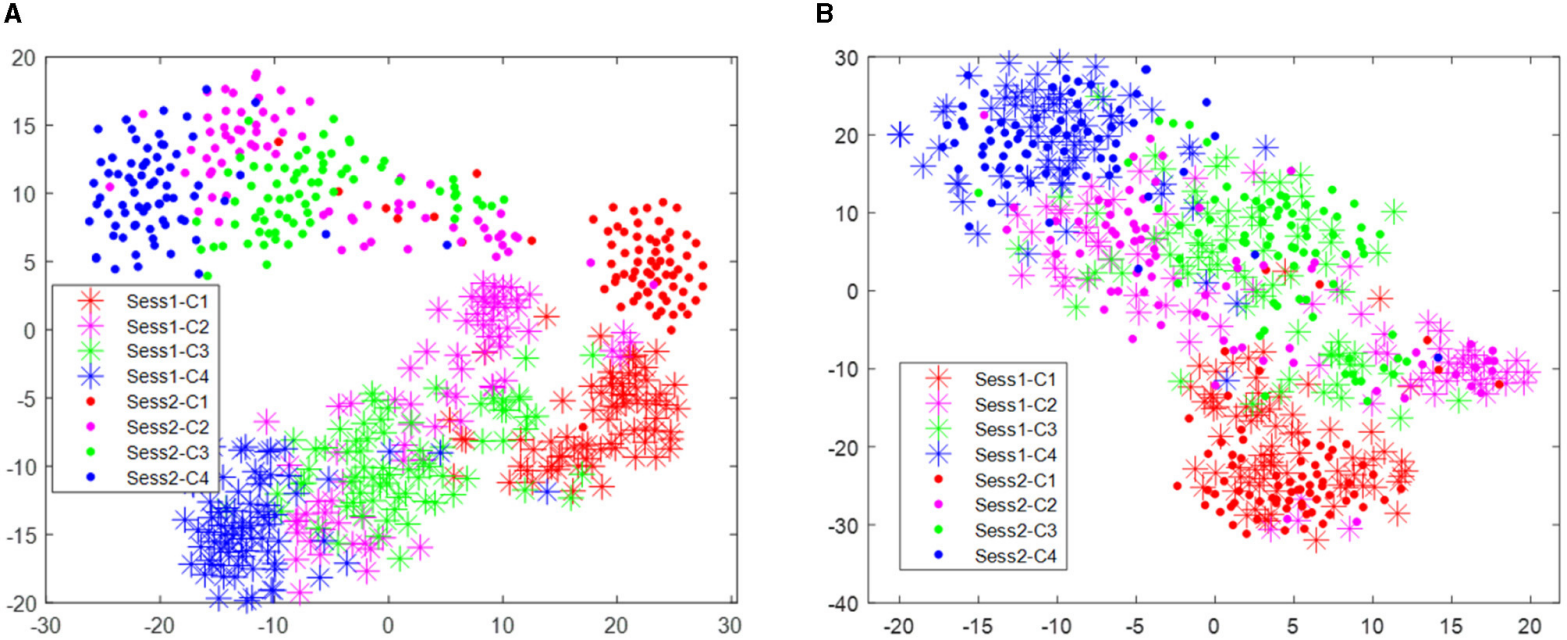
Distribution of the two sessions for subject 9 in the data set BCI IV 2a. Stars represent data points from session 1, while filled circles represent data points from session 2. Four different colors show four different classes. **(A)** Illustrates the distribution before transfer. **(B)** demonstrates the distribution after transfer by RPA-LEM.

The distribution improvement effects across all subjects are relatively similar. In this study, we have only displayed the comparison for one subject. Figure 2 displays the distribution of the two different sessions for subject 9 in the BCI IV 2a data set. From Figure 2, we can observe that the distribution of the two sessions are clearly different in the original feature space before RPA-LEM being applied. Even for the same subject, EEG signals collected on different dates exhibit significant distribution variations, making EEG signal recognition challenging. Classifiers trained on one session will not perform well in another session. However, after transfer via RPA-LEM, the distribution differences between sessions were noticeably reduced, which may significantly improve the performance of the classifier trained on one session but used on the other session.

**Figure 3:**
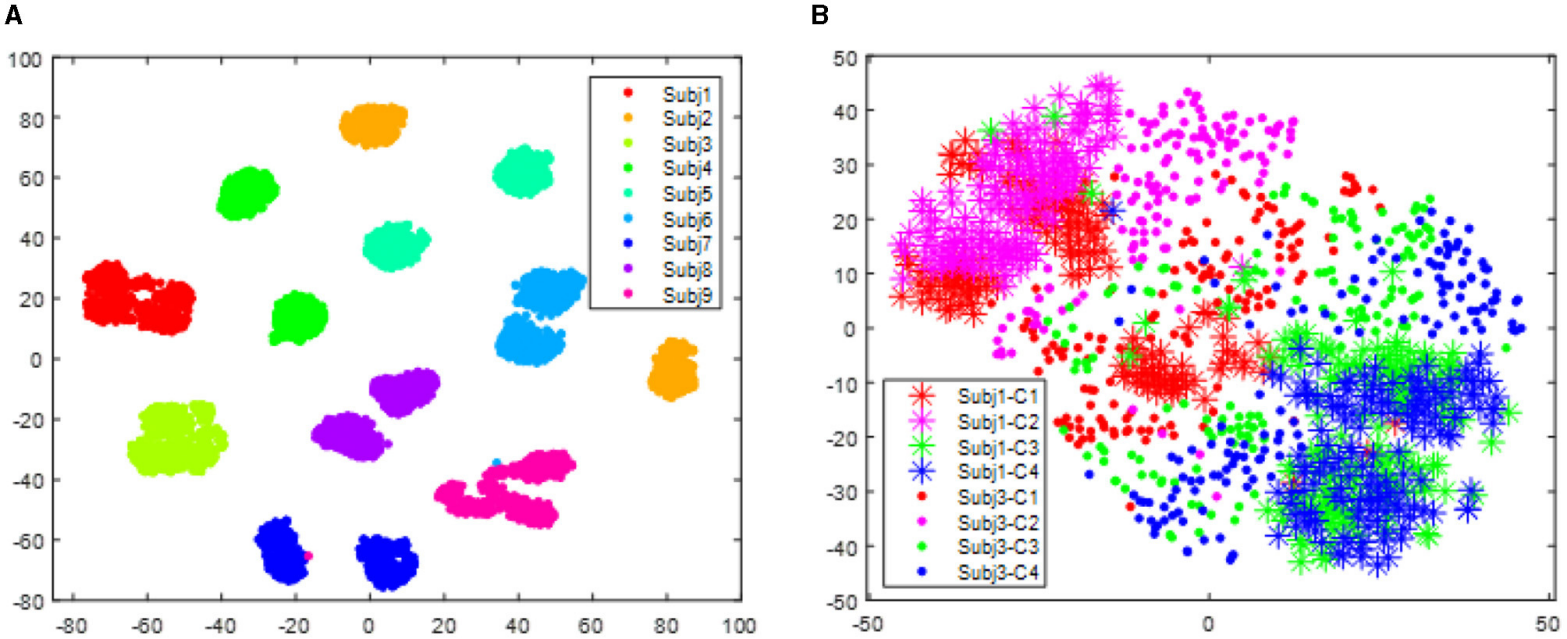
Distribution of data points for all subjects in the data set BCI IV 2a. **(A)** illustrates the distribution before transfer. Different color represents data comes from different subjects. **(B)** demonstrates the distribution of subject 1 and subject 3 after alignment by RPA-LEM.

Figure 3 visualizes the data from all subjects within the BCI IV 2a dataset, showing significant distribution differences among different subjects. These differences will make the classifier trained on one subject fail to obtain reasonable performance on another subject. However, after alignment via RAP-LEM, the distribution of data from subject 1 overlaps with that from subject 3, suggesting that RAP-LEM successfully matches the data from the two subjects. Thus, after alignment via RAP-LEM, the classifier trained on subject 1 will also give nice classification performance on subject 3.

**Figure 4:**
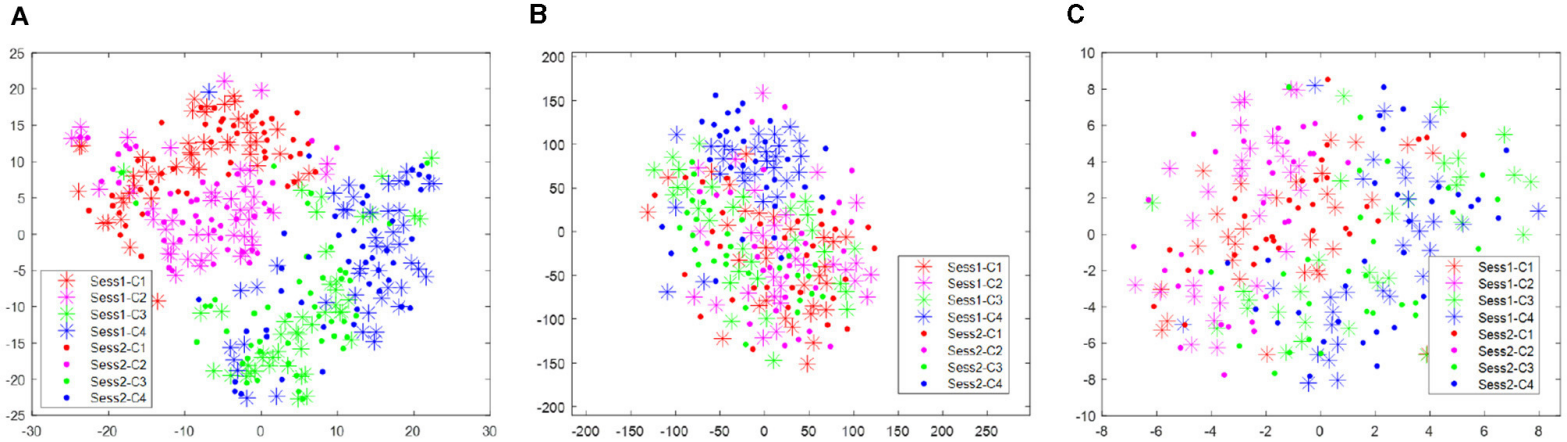
Distribution of the two “sessions” for the three subjects in the data set BCI IV 2a, respectively. Stars represent data points from session 1, while filled circles represent data points from session 2. Four different colors show four different classes. **(A)** illustrates the distribution for subject k3b. **(B)** demonstrates the distribution for subject k6b. **(C)** demonstrates the distribution for subject l1b.

**Figure 5:**
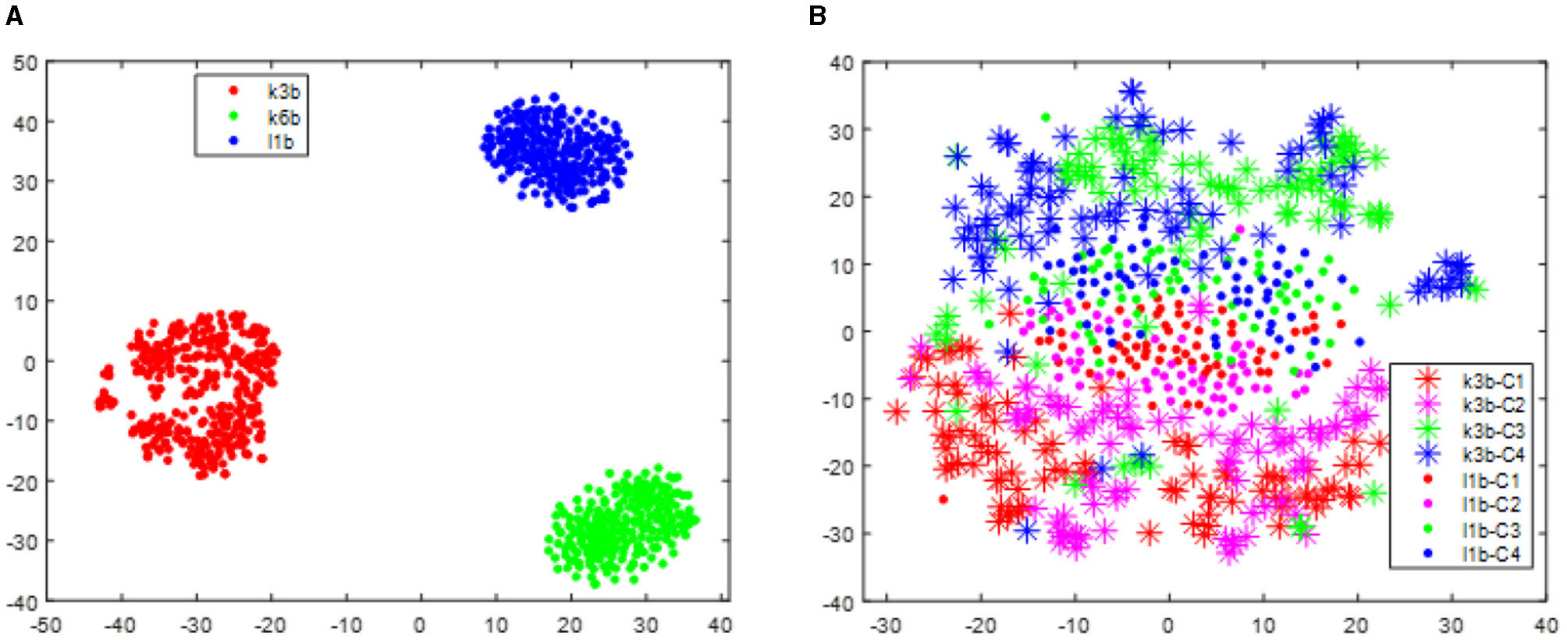
Distribution of data points for all subjects in the data set BCI III IIIa. **(A)** illustrates the distribution before transfer. Different colors represent data comes from different subjects. **(B)** demonstrates the distribution of subject k3b and subject l1b after alignment by RPA-LEM.

With respect to the data set BCI III IIIa, as the EEG signals of each subject were collected within the same day, no distribution differences were expected. However, to give a clear illustration, we visualized the data treating the predefined training split and test split as two different sessions. As shown in Figure 4, no apparent distribution difference between two “sessions” was observed. Consequently, RPA-LEM will not provide significant improvement on classification results. However, as shown in Figure 5, the distribution disparities between subjects are significant. After applying RPA-LEM, the distribution disparities between subjects (e.g., k3b and l1b) can be significantly reduced.

### 4.3 Model performance analysis

The performance of the proposed transfer learning approaches was evaluated on the BCI IV 2a and BCI III IIIa data sets using PLVQ-LEML as classifier. Then, we compared the performance of PLVQ-LEML with and without the proposed transfer learning approaches (PA-LEM and RPA-LEM) and the original RPA. We also compared the final performance with existing results in the literature.

#### 4.3.1 BCI IV 2a

We first evaluated our proposed transfer learning approaches in the cross-session transfer setting, which matches the data distribution of the given testing session with that of the given training session for each subject in the data set BCI IV 2a. The experiments were repeated for 10 runs. The averaged test kappa values over 10 runs is shown in Table 6. The Riemannian classifier PLVQ-LEML was employed. The term “no-transfer” means classification using PLVQ-LEML without any transfer operations applied. The results in the table are organized in two parts, with high-quality subjects at the top and low-quality subjects at the bottom. From Table 6, we can observe that the inclusion of the transfer learning approaches generally outperformed the no transfer approach. Our proposed RPA-LEM approach performed slightly better than RPA on average, while our proposed PA-LEM performed worse than RPA. In general, for high-quality subjects, our approach RPA-LEM can improve the performance by a rate as high as 11.14%, on average, compared with no transfer, while for low-quality subjects, our approach RPA-LEM can only improve that by a rate of 2.5%. The overall performance of our proposed RPA-LEM is comparable to that of RPA. The PLVQ-LEML with our proposed RPA-LEM (PLVQ-LEML + RPA-LEM) obtained much better results compared with existing results, as shown in Table 7, suggesting the effectiveness of the proposed method.

**Table 6:** Performance comparison between no-transfer and three transfer learning methods in cross-session transfer setting on the data set BCI IV 2a.

**Subject**	**No-transfer**	**RPA**	**PA-LEM**	**RPA-LEM**
S1	0.7870 (0.271)	0.8244 (0.004)	0.8009 (0.006)	**0.8250** (0.008)
S3	0.7963 (0.042)	**0.8537** (0.004)	0.8565 (0.011)	0.8503 (0.003)
S7	0.7176 (0.067)	**0.8105** (0.006)	0.8009 (0.07)	**0.8105** (0.008)
S8	0.6944 (0.091)	0.7728 (0.005)	0.7453 (0.021)	**0.7799** (0.008)
S9	0.7315 (0.070)	0.7596 (0.006)	0.7222 (0.009)	**0.7707** (0.006)
mean	0.7454	0.8042	0.7852	**0.8073**
S2	0.3241 (0.037)	**0.3306** (0.006)	0.3240 (0.008)	0.3293 (0.008)
S4	0.5370 (0.083)	0.5373 (0.006)	0.5046 (0.014)	**0.5417** (0.009)
S5	0.3750 (0.068)	0.3565 (0.011)	0.3564 (0.009)	**0.3596** (0.008)
S6	0.3333 (0.014)	**0.3802** (0.008)	0.3055 (0.008)	0.3799 (0.009)
mean	0.3924	0.4012	0.3726	**0.4026**

The best performance is marked in boldface.

**Table 7:** Performance comparison between our method and existing methods on the data set BCI IV 2a.

**Method**	**Mean kappa**	**S1**	**S2**	**S3**	**S4**	**S5**	**S6**	**S7**	**S8**	**S9**
PLVQ-LEML + RPA-LEM	**0.627**	0.83	0.33	0.85	0.54	0.36	0.38	**0.81**	**0.78**	0.77
MRGF-SVM (Xie et al., 2022)	0.616	0.83	0.46	0.78	0.53	0.32	0.39	0.79	0.76	0.68
Sharbaf et al. (2017)	0.61	0.75	0.31	0.82	0.56	0.47	0.38	0.75	0.74	0.67
Davoudi et al. (2017)	0.60	0.75	**0.49**	0.76	0.49	0.34	0.36	0.68	0.76	0.76
Gaur et al. (2018)	0.60	**0.86**	0.24	0.70	**0.68**	0.36	0.34	0.66	0.75	0.82
Luo et al. (2020)	0.60	0.63	0.17	**0.88**	0.38	**0.69**	**0.41**	0.76	0.76	0.69
Tang et al. (2021a)	0.59	0.79	0.32	0.76	0.55	0.34	0.36	0.69	0.71	**0.80**
Tang et al. (2021b)	0.59	0.75	0.34	0.80	0.58	0.38	0.37	0.70	0.64	0.75
1st (FBCSP)	0.57	0.68	0.42	0.75	0.48	0.40	0.27	0.77	0.75	0.61

The best performance is marked in boldface.

We then evaluated our proposed transfer learning approaches in the cross-subject transfer setting, treating data from one subject as training set and data from another subject as test set. To directly compare our methods with that is reported in the study by Zanini et al. (2018), which uses MDRM as classifier and RPA to reduce the distribution differences between the test subject and the training subject (MDRM+RPA), we followed their experimental setting that only utilizes the high-quality subjects (i.e., S1, S3, S7, S8, and S9). For each test subject, one subject among the remaining good subjects was used as the training subject. For each subject, the experiments were repeated until all the remaining good subjects were used as the training subject once. The mean kappa value together with standard deviation in bracket is shown in Table 8. From Table 8, we can observed that the incorporation of the Riemannian transfer learning significantly improved the classification performance. Our proposed RPA-LEM performed on par to the original RPA on this challenging task.

**Table 8:** Performance comparison between no-transfer and three transfer learning methods in the cross-subject transfer setting on the data set BCI IV 2a.

**Test subject**	**No-transfer**	**RPA**	**RPA-LEM**	**MDRM + RPA**
S1	0.5037 (0.16)	**0.6565** (0.07)	0.6561 (0.07)	0.6040 (0.08)
S3	0.5102 (0.17)	**0.7098** (0.04)	0.7050 (0.04)	0.6940 (0.04)
S7	0.4143 (0.13)	**0.5713** (0.10)	0.5577 (0.10)	0.5700 (0.09)
S8	0.4214 (0.05)	0.6162 (0.06)	0.6088 (0.06)	**0.6320** (0.07)
S9	0.4090 (0.09)	0.6382 (0.06)	0.6398 (0.07)	**0.6880** (0.06)
mean	0.4517	**0.6384**	0.6335	0.6376

The best performance is marked in boldface.

**Figure 6:**
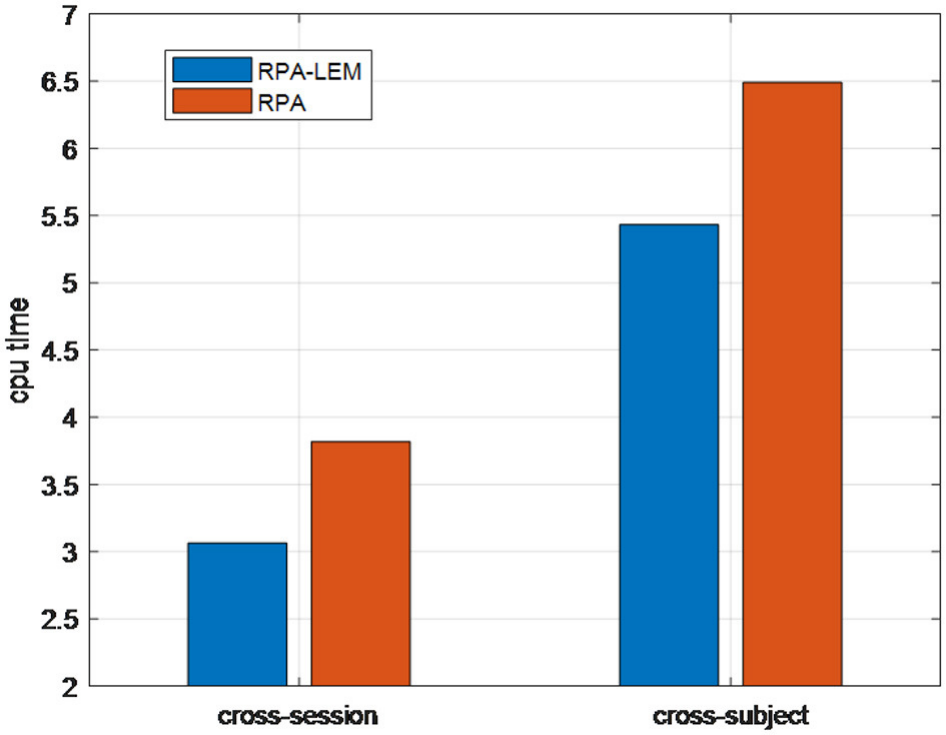
CPU time of RPA-LEM compared with RPA on the BCI IV 2a data set.

We finally compared the computational time (i.e., CPU time) of our proposed RPA-LEM with RPA. In BCI systems, real-time performance is crucial. Thus, when the classification performances are comparable, more computationally efficient methods are favorable. Figure 6 demonstrates the CPU time taken by our proposed RAP-LEM for the entire training and test process of one subject on the data set BCI IV 2a under both cross-session and cross-subject transfer settings. The reported CPU time is the averaged one over 10 runs, removing the randomness coming from the computing resource assignment. From Figure 6, we can observe that our proposed RPA-LEM is much more computationally efficient than RPA on both transfer settings.

In summary, on the BCI IV 2a data set, our proposed RPA-LEM obtained slightly better or comparable classification performance to RPA under both cross-session and cross-subject transfer settings, with much higher computational efficiency.

#### 4.3.2 BCI III IIIa

Similarly, we evaluated our proposed approaches in the cross-session transfer setting on the data set BCI III IIIa. The IIIa dataset is recorded in one single day. For cross-session evaluation, we split the dataset into two sessions in a random way. Repeated experiments of 10 times are conducted, and then, results are averaged to represent the experimental outcome. As Figure 4 illustrated, no apparent distribution differences were observed, since the data of each subject were recorded continuously at a single day. Thus, the performance of the classifier is not expected to be improved significantly by the transfer approaches. This is corroborated by our experimental results, as shown in Table 9. The performance of the classifier with transfer approaches remained similar to that without transfer. Again, our proposed RPA-LEM performed slightly better than our proposed PA-LEM but comparable to RPA. Additionally, PLVQ-LEML integrated with RPA-LEM obtained competitive performance to the existing methods, only seconded to the winner of the competition, as given in Table 10.

**Table 9:** Performance comparison between no-transfer and two transfer learning methods in the cross-“session” setting on the data set BCI III IIIa.

**Subject**	**No-transfer**	**RPA**	**PA-LEM**	**RPA-LEM**
k3b	0.9185 (0.080)	0.9200 (0.016)	0.9192 (0.012)	**0.9259** (0.019)
k6b	0.4556 (0.180)	0.4736 (0.015)	0.4593 (0.032)	**0.4778** (0.034)
l1b	0.7333 (0.075)	0.7411 (0.027)	0.7333 (0.023)	**0.7444** (0.019)
mean	0.7025	0.7116	0.7039	0.7160

The best performance is marked in boldface.

**Table 10:** Performance comparison between our method and existing methods on the data set BCI III IIIa.

**Method**	**Kappa**	**k3b**	**k6b**	**l1b**
1st	0.7926	0.8222	**0.7556**	**0.8000**
PLVQ-LEML + RPA-LEM	**0.7160**	**0.9259**	0.4778	0.7444
2nd	0.6872	0.9037	0.4333	0.7111
3rd	0.6272	0.9481	0.4111	0.5222
GLVQ-AIRM (Tang et al., 2021a)	0.6765	0.8519	0.4778	0.7000
MDRM	0.6222	0.8222	0.3556	0.6889
GLVQ	0.4481	0.5778	0.3444	0.4222
GMLVQ	0.3716	0.4815	0.1889	0.4444
GRLVQ	0.2654	0.1407	0.2444	0.4111

The best performance is marked in boldface.

**Figure 7:**
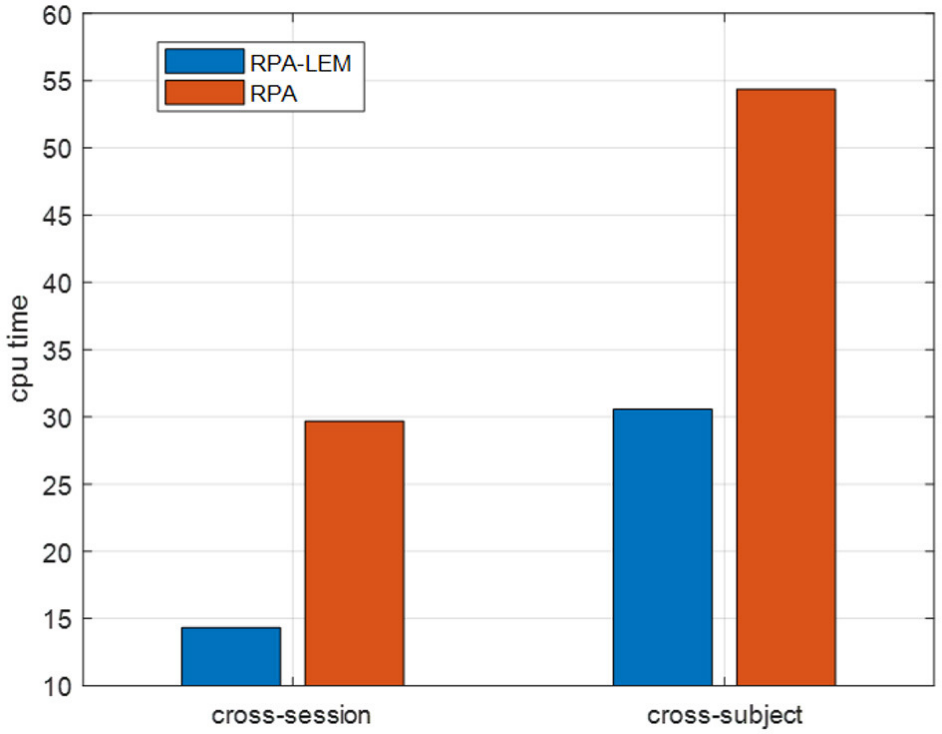
CPU time of RPA-LEM compared with RPA on the BCI III IIIa data set.

Again, we evaluated our proposed approaches in the cross-subject transfer learning setting on the data set BCI III IIIa. Similarly, for every test subject, each of the remaining subjects works as the training subject once. The averaged kappa values together with the standard derivation in brackets are reported, see Table 11. From Table 11, we can observe that the classification performance of our proposed RPA-LEM is comparable to RPA but significantly better than that of no-transfer. More importantly, our proposed method is much computationally efficient than RPA in both settings, as shown in Figure 7.

**Table 11:** Performance comparison of kappa between no-transfer and two transfer learning methods in the cross-subject transfer learning setting on the data set BCI III IIIa.

**Subject**	**No-transfer**	**RPA**	**RPA-LEM**
k3b	0.2772 (0.04)	**0.3230** (0.02)	0.3072 (0.04)
k6b	0.2306 (0.06)	0.3768 (0.10)	**0.3793** (0.12)
l1b	0.2085 (0.15)	**0.3814** (0.17)	0.3688 (0.14)
mean	0.2388	0.3604	0.3517

The best performance results among each row is marked in boldface.

## 5 Conclusion

This paper introduces two Riemannian transfer learning methods based on log-Euclidean metric termed as PA-LEM and RPA-LEM to reduce the distribution differences between the source data and the target data, both of which are points living on the Riemannian space of symmetric positive definite (SPD) matrices, aiming to improve the classification performance of the Riemannian classifier PLVQ-LEML efficiently on the target data. PA-LEM is equivalent to perform Euclidean Procrustes analysis (PA) in the logarithm domain of the SPD matrix-valued data. RPA-LEM adapts RPA, which computes Riemannian mean and dispersion of samples under computationally demanding affine-invariant Riemannian metric (AIRM), by log-Euclidean metric, yielding slightly better classification performance than RPA, with much higher computational efficiency.

Our methods are unsupervised transfer learning methods that do not require knowledge of labels of the target domain and thus can be used for online learning setting. One of our future work is to adapt our method for online learning setting, which learns the Riemannian mean and dispersion can be computed in an online fashion. In such scenario, when more experiments are conducted, the target domain data gradually increases, the obtained geometric mean will tend toward true value. Thus, the improvement in the classification performance will gradually increase over time. Even though the proposed Riemannian transfer learning approach in this study is initially designed for EEG data, it is actually applicable for any learning scenarios, where the inputs can be represented by SPD matrices. One typical example is the image set classification, where a set of images is represented by their covariance matrix. Thus, one of our future studies is devoted to explore the application of our proposed method in other learning scenarios, such as image set classification.

## Data availability statement

The original contributions presented in the study are included in the article/supplementary material, further inquiries can be directed to the corresponding author.

## Author contributions

FZ: Data curation, Formal analysis, Investigation, Methodology, Software, Validation, Writing—original draft, Writing—review & editing, Visualization. XZ: Conceptualization, Formal analysis, Methodology, Software, Validation, Visualization, Writing—original draft, Writing—review & editing. FT: Writing—review & editing, Funding acquisition, Project administration, Resources, Supervision, Conceptualization. YY: Writing—review & editing, Validation, Data curation. LL: Project administration, Writing—review & editing, Conceptualization.
